# Seabird Guano Fertilizes Baltic Sea Littoral Food Webs

**DOI:** 10.1371/journal.pone.0061284

**Published:** 2013-04-11

**Authors:** Karine Gagnon, Eva Rothäusler, Anneli Syrjänen, Maria Yli-Renko, Veijo Jormalainen

**Affiliations:** Section of Ecology, Department of Biology, University of Turku, Turku, Finland; University of Otago, New Zealand

## Abstract

Nutrient enrichment in coastal marine systems can have profound impacts on trophic networks. In the Baltic Sea, the population of Great Cormorant (*Phalacrocorax carbo sinensis*) has increased nearly exponentially since the mid-1990s, and colonies of these seabirds can be important sources of nitrogen enrichment for nearby benthic communities due to guano runoff. In this study we used stable isotope analyses and diet mixing models to determine the extent of nitrogen enrichment from cormorant colonies, as well as to examine any possible changes in herbivore diet preferences due to enrichment. We found significantly higher levels of δ^15^N in samples from colony islands than control islands for producers (the dominant macroalga *Fucus vesiculosus*, filamentous algae, and periphyton) and herbivores, as well as a positive correlation between enrichment and nest density in colony sites. We also found that enrichment increased over the breeding season of the cormorants, with higher enrichment in late summer than early summer. While the amount of total nitrogen did not differ between colony and control sites, the amount of guano-based nitrogen in algae was >50% in most sites, indicating high nitrogen enrichment from colonies. Herbivores (the isopod *Idotea balthica* and the gastropod *Theodoxus fluviatilis*) preferred feeding upon the dominant macroalga *Fucus vesiculosus* rather than on filamentous algae or periphyton in both control and colony, and there was a significant increase in periphyton consumption near colony sites. Overall, guano from cormorant colonies seems to have effects on both producers and herbivores, as well as the potential to modify algae-herbivore interactions.

## Introduction

Coastal seas are facing numerous threats to biodiversity and ecosystem functioning, such as eutrophication, overfishing, and the introduction of non-native species [1], [2], [3], [4], [5]. Traditionally, studies have focused on the impacts of a single stressor on a target species; however, recent studies have shown that examining multiple disturbances on the entire ecosystem is necessary to determine the full impacts of anthropogenic stressors. Trophic links between species can be very strong, such that changes in the abundance of one species can affect the whole trophic network through either top-down or bottom-up effects, with unexpected and far reaching impacts on the entire community [6], [7].

In the Baltic Sea, multiple disturbances [8], have already led to shifts in marine communities, e.g. [9], [10]. Among the more recent developments is the return of the Great Cormorant (*Phalacrocorax carbo sinensis*), which disappeared from northern Europe in the 19^th^ century [11], and returned to the Baltic Sea in the mid-1990s [12]. While the initial population growth was extremely rapid, the Great Cormorant population on the Finnish coast is starting to stabilise at approximately 17 000 breeding pairs (unpublished monitoring data from the Finnish Environmental Institute in 2012).

Cormorants form dense colonies (up to 2000 pairs) on islands and it has been suggested that these islands can be considered as small-scale models of highly eutrophicated, and depleted communities for several reasons [13], [14]. Due to their position at the top of the marine food chain, cormorants, like other seabirds, excrete guano which contains 15–20% nitrogen [15], as well as phosphorus [16], [17], [14]. Guano accumulates on colony islands during the breeding season (May-August in the Baltic Sea), and if washed into the sea can cause nitrogen (N) enrichment in the surrounding areas [14]. The extent of enrichment may also vary depending on the amount of runoff due to e.g. seasonal changes in guano accumulation and rainfall.

Cormorants are effective predators and consume large amounts of fish during the breeding season [18], [19], [20], [21] thus not just producing guano on colony islands but also generating top-down cascading effects. While top-down effects are often strong in marine littoral benthic systems [22], [23], [24], cormorant colonies also have the potential to considerably affect trophic interactions through bottom-up effects. Seabird colonies have been shown to lead to increased N concentrations in terrestrial plants and marine organisms [17], [25], [26]. N enrichment in benthic systems can generally increase the productivity of these ecosystems by stimulating the growth of algae [27], though not all algal species are equally affected. Fast-growing ephemeral filamentous algae benefit the most from N enrichment, while slower-growing perennial macroalgae are disadvantaged due to overgrowth of the filamentous algae and periphyton [27], [28]. Herbivory, at least at moderate levels, may counteract these effects, by reducing the coverage of filamentous algae [27]. Consequently, if N enrichment (i.e. eutrophication) is accompanied by trophic cascades (i.e. from overfishing or cormorant predation), which lead to higher herbivore densities, the top-down effects could balance the bottom-up effects. Therefore, islands inhabited by cormorants offer an important opportunity to explore the relationship and role of both bottom-up and top-down processes in regulating trophic interactions.

Prior to determining the interactions between processes, the magnitude and characteristics of cormorant impacts must be studied. N enrichment does occur in benthic communities near cormorant colonies on the Swedish coast of the Baltic Sea [14], however little work has been done in determining the factors controlling the spatial and temporal variability of guano enrichment. In this study, we determined the extent of N enrichment at producer and herbivore trophic levels near cormorant colonies on the Finnish coast, and the role of nest density and fetch in influencing enrichment. We predicted that high nest densities and high wave exposure (i.e. fetch) would have a positive effect on N enrichment around colonies. We also tested how enrichment varied seasonally, as well as whether guano-based N affected control islands, with the hypothesis that enrichment is a solely local scale phenomenon which increases over the summer (along with higher guano production and increasing rainfall). In addition, we applied diet mixing models to estimate the contribution of guano-based N to N uptake of algae and periphyton, and to determine the diet preferences of herbivores and their possible shifts due to N enrichment from guano. We predicted that the consumption of periphyton and/or filamentous algae would increase in colonies as compared to control islands due to positive impacts of nitrogen enrichment on algal growth.

## Materials and Methods

### Ethics statement

All sampling complied with relevant regulations. No permits were required as sampling occurred outside the cormorant breeding season and no protected species were sampled.

### Study area and cormorant colonies

**Figure 1:**
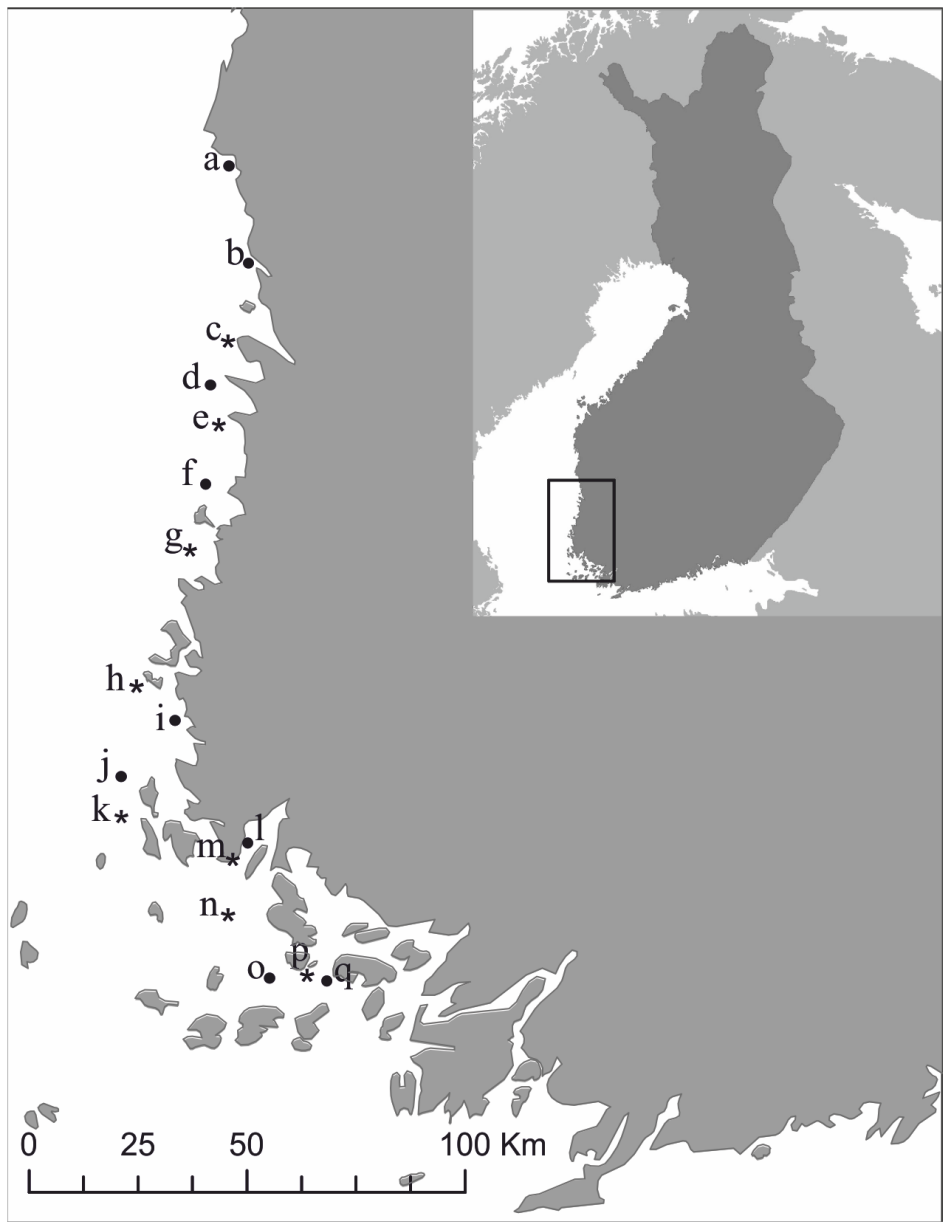
Locations of colony (circles) and control (stars) sites along the southwestern Finnish coast. The letters refer to sites listed in Table 1.

Our study sites represent hard-bottom, non-tidal littoral habitats along about 200 km of the Finnish coastal southwestern archipelago (Figure 1). These habitats are typically dominated by *Fucus* assemblages with *Fucus vesiculosus* as the foundation species and *Cladophora spp*., *Ulva* spp., *Ectocarpus siliculosus*, *Pilayella littoralis*, *Ceramium tenuicorne* and *Furcellaria lumbricalis* as typical but by no means the only associated macroalgae. Invertebrate fauna is dominated by gastropods (mostly *Hydrobia* spp. and *Theodoxus fluviatilis)*, isopods (especially *Idotea* spp.), and gammarid amphipods. Our study sites include eight colony islands with varying areas and cormorant populations, and eight respective control islands (Table 1). Although cormorants dominate the avian fauna on colony islands, several other seabirds such as gulls, terns, razorbills, guillemots as well as grey herons also nest associated with the colonies (personal observation).

**Table 1:** Summary of colony and control site characteristics, including fetch, breeding pairs, area, and year of colony establishment (for colony islands).

Code	Site	Colony/Control	Average Fetch (km)	Maximum Fetch (km)	Breeding Pairs (2011)	Area (ha)	Year of colony establishment
a	Lankoslahti	Colony	0.31	0.58	1619	1.06	2002
b	Järvikari	Colony	19.23	57.68	1252	0.69	2005
d	Marjakari	Colony	22.81	64.19	1084	1.93	2003
f	Puskakarta	Colony	23.32	70.42	2342	3.64	2005
i	Urpoinen	Colony	0.81	1.66	190	4.18	2010
j	Kluppi	Colony	1.79	4.99	417	5.18	2003
l	Kalmanhohde	Colony	1.52	3.27	241	0.18	2009
o	Äggskär	Colony	1.37	2.67	2000	2.42	2003
q	Måsgrund	Colony	1.59	3.09	520	0.3	2009
c	Outoori	Control	9.91	30.81			
e	Matinkari	Control	6.92	21.06			
g	Kuuskajaskari	Control	5.72	16.25			
h	Päiväkarit	Control	5.72	17.12			
k	Korra	Control	6.71	20.75			
m	Raumharju	Control	0.47	1.08			
n	Mustaluoto	Control	0.68	1.23			
p	Orhisaari	Control	1.65	3.74			

The average and maximum fetch are given for the actual sampling point from each island. Data from the colony islands were provided by the Finnish Environment Institute, which monitors cormorant colonies.

The study area is characterized by thousands of islands and islets of varying size and exposure to waves, the latter related to wave energy and exchange of water-masses. To estimate wave exposure for each study site we derived the fetch values (Table 1) from the database generated by the Laboratory of Computer Cartography, University of Turku. The database includes average and maximum fetch values calculated for points at 10-m intervals of shorelines in our study area [29].

### Sampling

The guano of fish-eating seabirds typically has highly enriched δ^15^N ratio due to their high trophic position; it therefore provides a distinctive signature of seabird-based nitrogen (N) in food webs. To detect and estimate the amount of N in the food web originating from cormorant colonies we sampled producers (*F. vesiculosus*, *Cladophora glomerata*, *Ulva* spp., and periphyton) and consumers (*Theodoxus fluviatilis*, *Idotea balthica*) from eight colony islands and eight control sites (Table 1, Figure 1). Not all species were present at all sites; the two northernmost colony islands (marked by *a* and *b* in Figure 1) had neither *F. vesiculosus* stands nor *I. balthica*, and the colony island *b* also did not have the gastropod *T. fluviatilis*. Colony island *i* did not have *I. balthica*, while colony island *q* did not have any *F. vesiculosus* or *C. glomerata*. Finally, the control islands *e* and *m* were missing *Ulva* spp. and *C. glomerata*, respectively.

We collected samples in September 2011 by snorkeling in the shallow (<3 m) infralittoral within an area of <1000 m^2^ in a random spot around each island (topography of the location was not considered, though it may have an influence on guano runoff from colonies [14]). For periphyton samples, three individuals of *F. vesiculosus* were detached from their substratum. Each thallus was then individually rinsed in seawater, which was filtered through a 200- µm mesh-net to remove larger particles. Subsequently, periphyton was collected from the bottom of the filter. In addition, three samples of both *Ulva* spp. and *C. glomerata*, and five individuals of *F. vesiculosus* were collected from distinct locations within the snorkeling area. From each *F. vesiculosus* individual, we took three samples from different parts of the thallus: 7–9 cm (hereafter base), 3–4 cm (hereafter mid), and 0–1 cm (hereafter tip) from the tip. Based on the knowledge of the growth rate of *F. vesiculosus* in the area [30], we could estimate the time when different parts of the thallus had grown; the base had grown at the time of cormorant egg-hatching in May, the middle at the time of large nestlings in July, and the tip after the nesting period in August-early September. All algal samples were cleaned to remove epibionts before they were blotted dry and preserved in silica gel until oven-drying in the laboratory at 55°C for 24 h.

Samples of *T. fluviatilis* and *I. balthica* were collected from *F. vesiculosus* or other macrophytes when *F. vesiculosus* was not present. At least 12 individuals of both species were sampled. The animals were kept in water without food for about 24 h to empty their gut contents and then preserved on ice until freezing within 1 to 4 days. In the laboratory, animals were oven-dried (as above) and ground to a fine powder. Four individuals were ground to form one composite sample and three composite samples of both species from each site were prepared. For the gastropod *T. fluviatilis* we only used the soft parts, detached from the shell, so that the isotope signal would only indicate accumulation during the present year.

To estimate the contributions of colony-based and other water-borne stable isotope signals to the food web, we took three samples of guano from each colony and three samples of surface water particulate organic matter (POM) from a distance of approximately 200 m from each control island. The δ^15^N in POM was assumed to represent the N available for producers in the water column without terrestrial runoff from seabird guano. The POM sample was taken by filtering 1 L of seawater through a 50- µm filter into a bottle that was kept on ice until vacuum-filtration of POM through GF/F Whatmann glass-fibre filters.

### Stable isotope analysis

The stable isotope analyses of ^13^C and ^15^N were conducted from dried samples (prepared as indicated above) in the UC Davis Stable Isotope Facility (University of California) using PDZ Europa ANCA-GSL elemental analyzer interfaced to a PDZ Europa 20-20 isotope ratio mass spectrometer (Sercon Ltd., Cheshire, UK). Analyses of the samples were run along with calibrated laboratory standards (nylon, bovine liver, glutamic acid and peach leaves) that were chosen to be compositionally similar to the samples being analyzed. The isotope values presented here are expressed as standard delta values relative to international standards of Vienna PeeDee Belemnite for carbon and atmospheric N for nitrogen as follows:
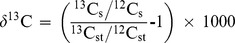


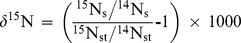
 where *s* refers to sample and *st* to standard.

The total amount of N content including all isotopes was also measured to determine whether it varied between colony and control islands.

Values of δ^13^C were corrected for variation in lipid content, which would otherwise generate unwanted variation for the carbon signal, by using the normalization technique suggested by Post et al. [31]. The correction for aquatic invertebrates uses the relative abundance of C to N as follows:
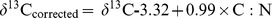



The correction for plants with less than 40% C (as algae and periphyton in our samples) uses the percentage of C as follows:
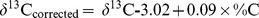



### Statistical analyses

For analysis of δ^15^N and δ^13^C signatures, as well as total N content, we used linear mixed models implemented by SAS 9.3 [32]. In separate analyses for both the signatures and all sample types, we used the treatment (colony/control) as the fixed factor and the site, nested within the treatment, as the random factor. We checked whether there was spatial autocorrelation between sites, and found no evidence of this, so location was not considered in the analysis. Due to heteroscedasticity of variances between the treatment levels (i.e. variances were higher within colony than control sites), we used a likelihood-based (REML estimation) statistical model which allowed us to fit different variance levels for each treatment level [32]. Thus, the model weighed data differently depending on the variance. We used the Akaike information criterion to judge that the model fitted the data better than a homogenous variance model. To determine whether the δ^15^N signature (i.e. enrichment) varied between different sections of the *F. vesiculosus* thallus, we used a model in which the sections were treated as repeated measures within the individual alga. Univariate linear regression analyses were used to determine the relationship between enrichment and abundance/density of breeding pairs within the colony sites, as well as to determine whether the fetch or wave exposure of the colony site affected enrichment.

### Applying diet mixing models

We applied diet mixing models for two purposes: 1) to perform a source analysis on producers and estimate the amount of N originating from the terrestrial runoff from colonies, and 2) to determine any possible differences in diet composition of consumers between colony and control islands. To carry out the diet mixing analyses, we used the SIAR V4 package for R, which uses Bayesian inference (see [33] for more details).

We used δ^15^N values from two sources for the producer source analysis: guano and POM alone. For each colony site we used the POM values from the nearest control site, and for each control site we likewise used the guano values from the nearest colony site. Only δ^15^N was used, as the carbon from the terrestrial runoff is unlikely to compete for intake by the producers. We also assumed no fractionation was taking place (i.e. trophic enrichment factor (TEF) = 0). The median value of guano uptake was taken to represent each site (see [33] for more details) and the mean of these medians was calculated for each species. We then used univariate linear regression analyses to determine whether nest density and fetch of colony islands affected the amount of guano-based N absorbed by producers.

Both δ^15^N and δ^13^C were used for the herbivore diet analysis, with three sources: *F. vesiculosus*, filamentous algae, and periphyton. *Ulva* spp. and *C. glomerata* were pooled as filamentous algae due to the similarity of their stable isotope signatures. We calculated the diet-dependent TEF separately for each source as recommended by Caut et al. [34]:
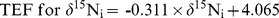


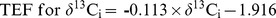
 where i refers to the diet (source).

TEFs were calculated using data from the control islands only, as we assumed these were more representative of the “natural” enrichment factors in this system. Since diet mixing models are especially sensitive to different enrichment factors, we also ran the same models using fractionation factors derived from a review involving several different systems [35], as well as those from two recent studies on pelagic marine ecosystems [36] and lake ecosystems [37]. Using these values, the patterns were similar and we only show the analyses based on enrichment factors calculated following Caut et al. [34]. Trophic fractionation factors used for the models are shown in Table 2.

**Table 2:** Trophic enrichment factors (mean ± standard deviation) of δ^15^N and δ^13^C from three diet sources used in the diet mixing analyses.

Source	TEF for δ^15^N	TEF for δ^13^C
Filamentous algae	2.15±0.62	0.26±0.33
Periphyton	2.36±0.38	0.34±0.23
*Fucus vesiculosus*	2.05±0.45	−0.4±0.29
McCutchan et al. [35]	2.3±0.28	0.4±0.17
Aita et al. [36]	3.5±0.2	0.9±0.39
Syväranta et al. [37]	3.0±0.5	0.5±0.2

These factors were calculated using the equations suggested for invertebrate consumers in Caut et al. [34]. Average from colony sites and standard deviation are shown. Also shown are trophic enrichment factors from three previous studies which were also used to compare model results. In these cases, the same enrichment factors were used for the three source species.

Since stable isotope signatures varied by site, each site was analysed separately with site-specific source values. Only sites in which all four source species were present were used. The median for each site was then estimated using SIAR and the mean of these medians calculated for each species and source. These means were then subsequently explored in linear mixed models [see [33]) to determine if there were significant differences between sources, within sites, and between colony and control sites. To account for interdependency between the sources, they were treated as repeated measures within each site.

## Results

### Stable isotope analysis

**Figure 2:**
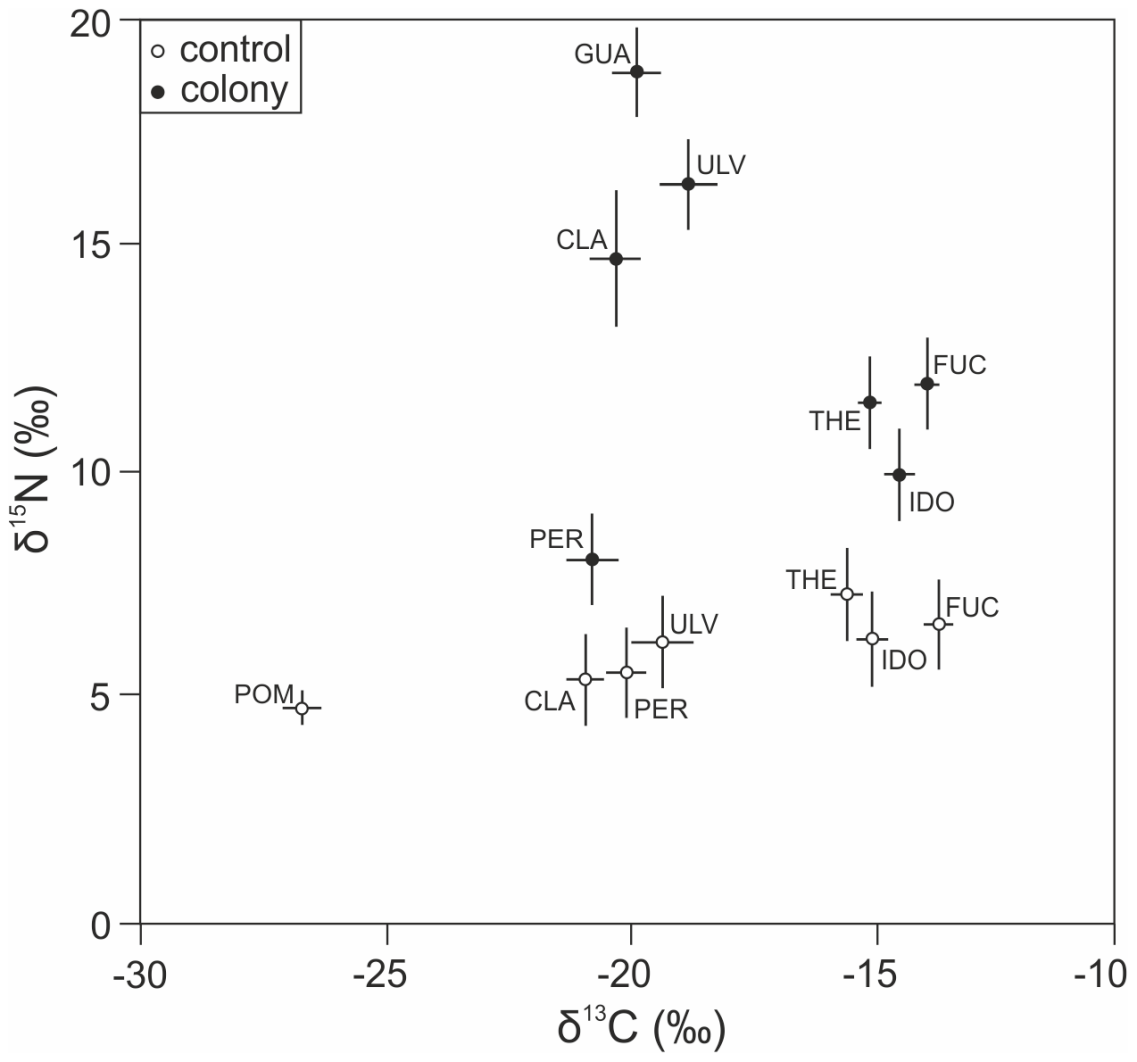
Stable isotope signatures (mean±SE) of all samples from colony (black circles) and control sites (white circles). POM  =  particulate organic matter (control sites only), GUA  =  guano (colony sites only), FUC =  *Fucus vesiculosus*, CLA =  *Cladophora glomerata*, ULV =  *Ulva* spp., PER =  periphyton, IDO =  *Idotea balthica*, THE =  *Theodoxus fluviatilis*.

The δ15N values were significantly higher in colony sites than in control sites for all species (Figure 2, Table 3). The mean, as well as the variance, in δ15N was much higher among the colony sites (*F. vesiculosus*: σ*^2^* = 36.64, *C. glomerata*: σ*^2^* = 52.73, *Ulva* spp.: σ*^2^* = 85.60, periphyton: σ*^2^* = 10.52, *I. balthica*: σ*^2^* = 11.96, *T. fluviatilis*: σ*^2^* = 8.92) than among the control sites (*F. vesiculosus*: σ*^2^* = 1.78, *C. glomerata*: σ*^2^* = 3.22, *Ulva* spp.: σ*^2^* = 3.93, periphyton: σ*^2^* = 1.50, *I. balthica*: σ*^2^* = 2.66, *T. fluviatilis*: σ*^2^* = 0.41). The total N content did not differ between colony and controls sites in any of the species. However, the δ15N increased significantly with total N content in *F. vesiculosus (β*_control_ = 1.27±0.36, r^2^_control_ = 0.22, P_control_ = 0.001; *β*_colony_ = 9.75±2.75, r^2^_colony_ = 0.28, P_colony_ = 0.001) and *Ulva* spp. (*β*_control_ = 1.08±0.38, r^2^_control_ = 0.26, P_control_ = 0.01; *β*_colony_ = 4.02±0.99, r^2^_colony_ = 0.35, P_colony_<0.001), particularly in the colony sites. We found no differences in the δ^13^C values between the colony sites and control sites in any of the species (Figure 2, Table 3).

**Table 3:** Summary of mixed ANOVAs showing the effects of colony islands on δ^15^N and δ^13^C values for different species.

	δ^15^N				δ^13^C			
Species	df (num)	df (den)	F	P	df (num)	df (den)	F	P
*Fucus vesiculosus*	1	11.7	7.01	**0.0217**	1	12	0.07	0.7926
Periphyton	1	16.2	7.80	**0.0129**	1	14	0.29	0.5972
*Ulva* spp.	1	13.9	7.69	**0.0150**	1	14	0.09	0.7631
*Cladophora glomerata*	1	13	9.99	**0.0075**	1	13	0.28	0.6039
*Idotea balthica*	1	10.8	7.33	**0.0206**	1	10.9	0.94	0.3531
*Theodoxus fluviatilis*	1	13.2	9.25	**0.0093**	1	14.1	0.90	0.3595

Statistically significant values are indicated in bold. Note that each species was analysed separately. For *Fucus vesiculosus*, the average for each individual (as calculated from the three thallus sections) was used.

There were significant differences between species within the colony sites for both δ^15^N (F_6, 159_ = 18.52, P<0.001), and δ^13^C (F_6, 159_ = 31.81, P<0.001). The δ^15^N value was highest in guano samples, followed by the filamentous algae, *F. vesiculosus*, herbivores, and finally periphyton, which had the lowest δ^15^N values. The δ^13^C values separated into two groups: *F. vesiculosus*, *I. balthica*, and *T. fluviatilis* were similar, and higher than those of *C. glomerata*, *Ulva* spp., and periphyton, while the δ^13^C values of guano resembled those of filamentous algae. Within the control sites, there was also some variation between the species for δ^15^N (F_6, 160_ = 14.13, P<0.001), though the differences were much smaller, and the pattern was different than in the colony sites: the δ^15^N value was highest in the herbivores and *F. vesiculosus*, followed by the filamentous algae and periphyton, and lowest values were found in the POM samples. The δ^13^C values also varied significantly between species in the control sites (F_6, 162_ = 161.01, P<0.001), along the same pattern as seen within the control sites with the addition of POM, which had a δ^13^C value that was clearly lower than that of all other samples.

**Figure 3:**
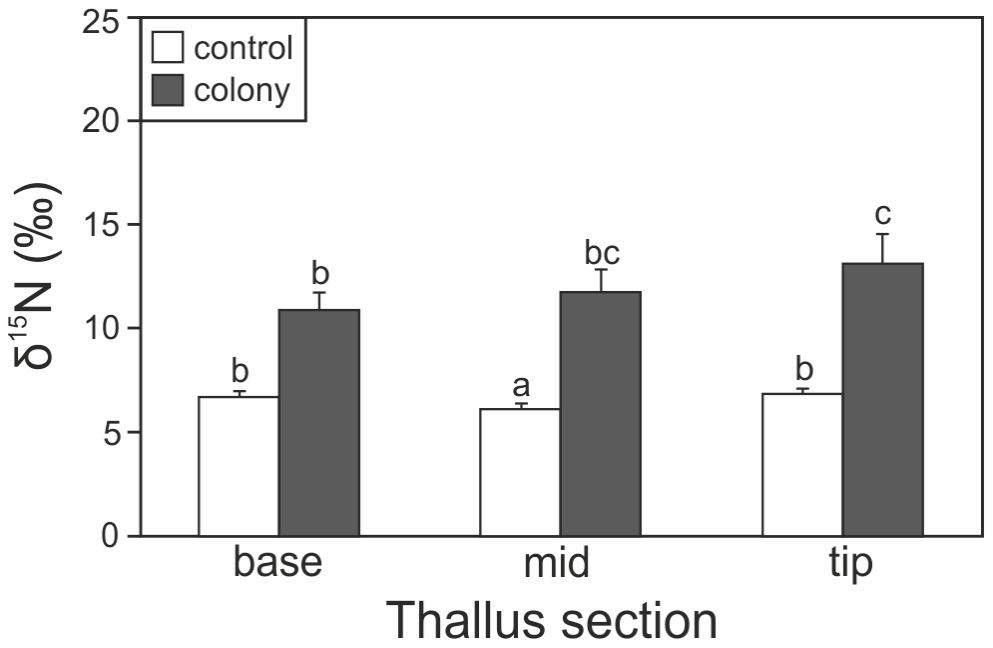
δ^15^N values (mean ± SE) of different sections of *Fucus vesiculosus* thalli from colony (black bars) and control (white bars) sites. Different letters indicate significantly different values within control or colony sites.

In *F. vesiculosus*, in addition to an overall significant difference between colony and control sites (Table 3), there was a significant interaction between treatment and thallus section for the δ^15^N value (F_2, 100_ = 4.09, P = 0.02). δ^15^N increased significantly from the base to the tip in individuals from colony islands, while there was no such trend in individuals from control islands (Figure 3), indicating an increased availability of N run-off from the colonies with the proceeding season. The difference between colony and control sites was not significant for the base section (P = 0.07), and then increased throughout the summer (mid section: P = 0.02; tip section: P = 0.01). There were no differences in δ^13^C between the different sections of the thallus or between colony and control sites.

**Figure 4:**
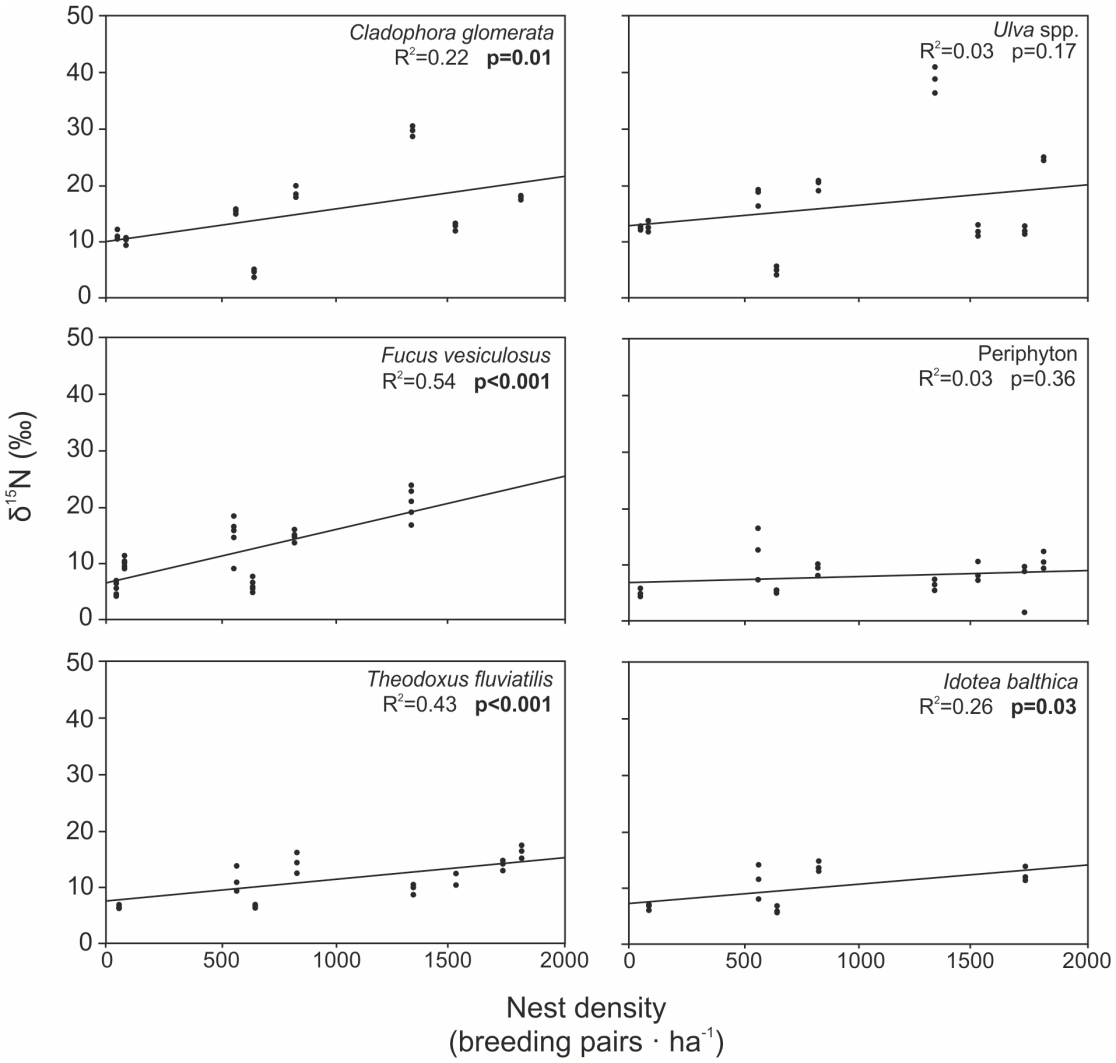
Effect of cormorant nest density on δ^15^N values for all species from colony sites. Significant regressions are shown in bold.

Within the colony sites, δ^15^N was positively correlated with the nest density for all species except *Ulva* spp. and periphyton (Figure 4). There were no significant relationships between either total or average fetch and δ^15^N within the colony sites (all r^2^<0.08, all P:s>0.6).

### Source and diet analyses

**Figure 5:**
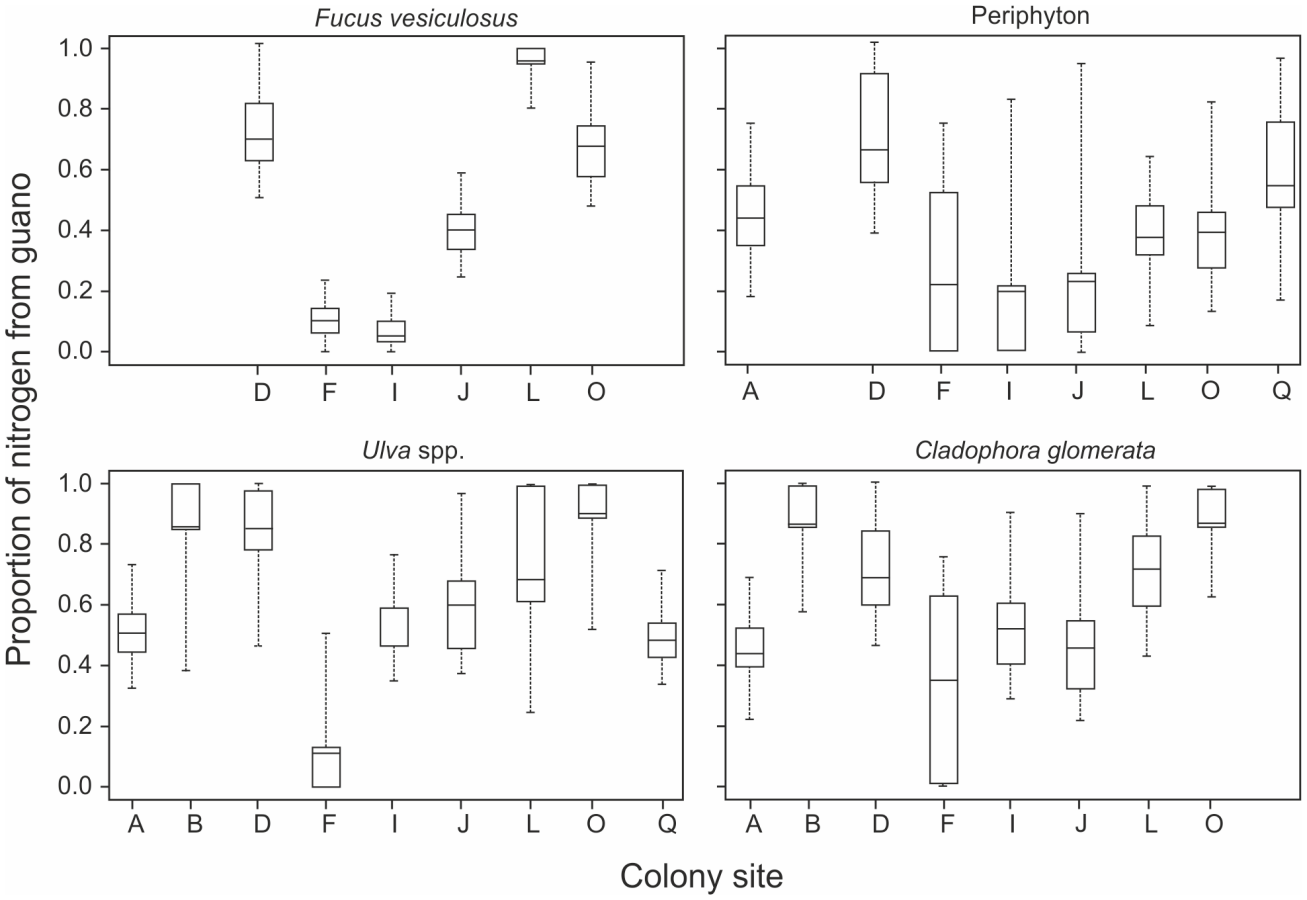
Proportion of nitrogen uptake from cormorant guano in algae and periphyton from colony sites. Median, 25%, 75%, 5%, and 95% credibility intervals are shown. Letters refer to sites listed in Table 1.

Algae and periphyton in the control islands showed some N uptake from guano (*F. vesiculosus*: mean proportion of guano = 0.14±0.02; *C. glomerata*: mean = 0.17±0.02; Ulva *spp*.: mean = 0.21±0.02; periphyton: mean = 0.15±0.01). There was no effect of fetch on the proportion of guano-based N in colonies, but a slight positive trend with nest density for *F. vesiculosus* (r^2^ = 0.43, P = 0.09). In algal species and periphyton from colony sites, however, the amount of N originating from guano comprised a large proportion of the N uptake (*F. vesiculosus*: mean proportion of guano = 0.48±0.14; *C. glomerata*: mean = 0.61±0.06; Ulva *spp*.: mean = 0.60±0.07; periphyton: mean = 0.42±0.05). However, the proportion of N uptake from guano varied considerably between the colony sites (Figure 5).

**Figure 6:**
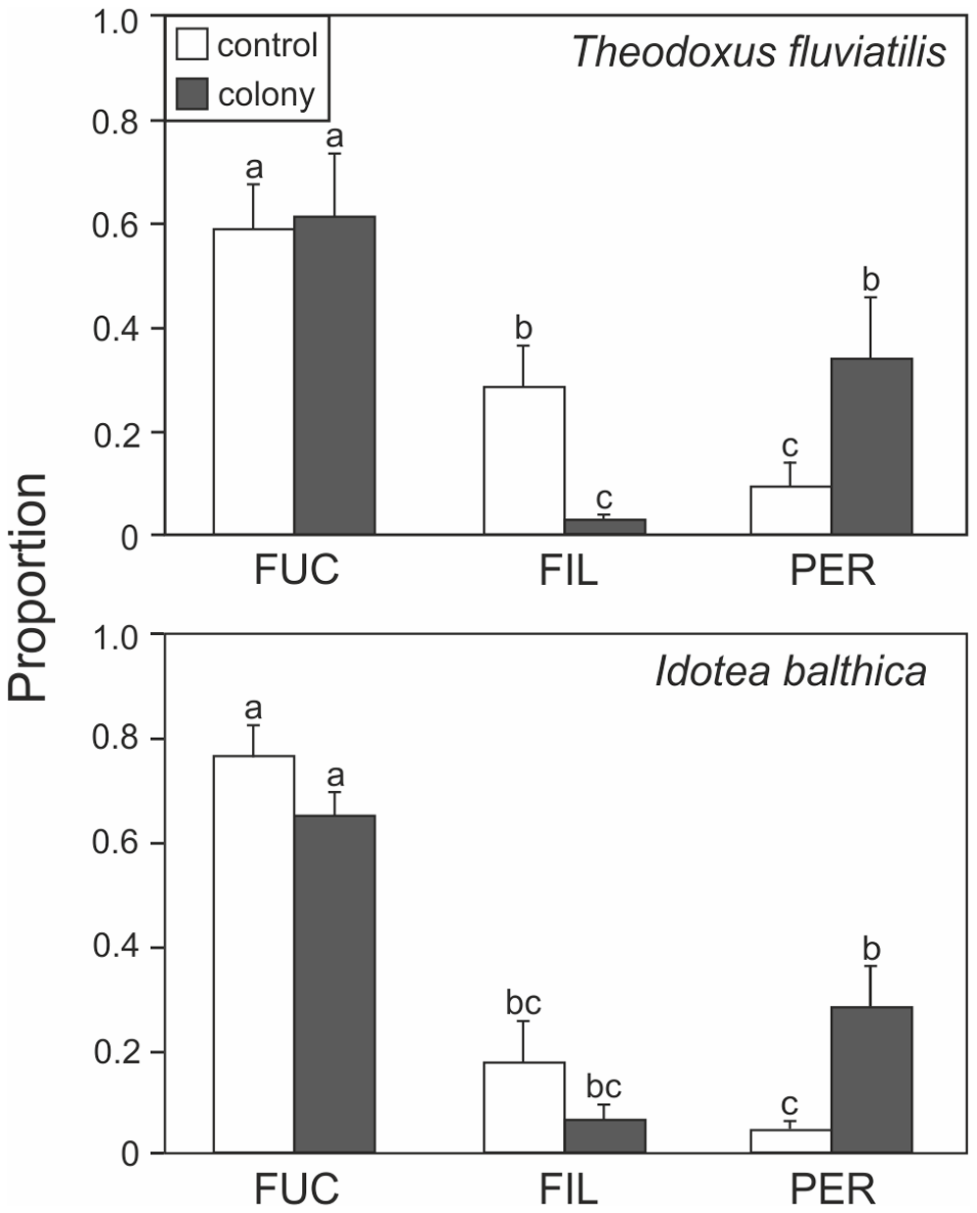
Proportion of sources (mean ± SE) from diet analysis of *Idotea balthica* and *Theodoxus fluviatilis* from colony and control sites. Results shown are based on diet mixing analysis using diet-dependent trophic fractionation factors following Caut et al. [34], given in Table 2. FUC =  *Fucus vesiculosus*, FIL =  filamentous algae (*Cladophora glomerata* and *Ulva* spp.), PER =  periphyton.

For the diet analysis of *T. fluviatilis and I. balthica*, we used three food sources: *F. vesiculosus*, filamentous algae (*Ulva spp*. and *C. glomerata*), and periphyton (Figure 6). There was a significant interaction between treatment (colony or control) and diet source for both species (*T. fluviatilis*: F_2, 30_ = 4.08, P = 0.03, *I. balthica*: F_2, 21_ = 6.61, P = 0.006), indicating that thefeeding patterns of grazers were modified by the nutrient enrichment from the colonies. For the gastropod *T. fluviatilis*, *F. vesiculosus* made up the highest proportion of the diet in both colony and control sites (and did not differ significantly between treatments), while the proportion of filamentous algae was significantly lower in colony than control sites, and the proportion of periphyton was significantly higher in colony than control sites (Figure 6). The pattern was similar for the isopod *I. balthica*: the proportion of *F. vesiculosus* was high and did not differ between control and colony sites. However, there was a slight but not significant decrease in the proportion of filamentous algae, and a significant increase of periphyton in colony sites as compared to control sites (Figure 6).

## Discussion

Our results show considerable N enrichment from cormorant colonies into surrounding benthic communities, as the δ^15^N values for all tested species were significantly higher in samples from colony sites as compared to control sites. Guano runoff affected producers and their herbivores to a similar extent, thus raising the possibility for effects at higher trophic levels such as predatory fish. However, effects on algae and invertebrates seemed to be rather localised. Most control islands were between 5–10 km from the colony islands, and even in cases where the distance was <5 km, there were significant differences between δ^15^N values. However, the source analysis revealed that some guano-based N is present in algae from control sites, but in clearly lower amounts than in colony sites. This guano-based N in control sites most likely originates from locally nesting and/or feeding seabirds (e.g. gulls, terns, eiders, and other waterfowl), which are abundant throughout the study area, but some guano may also have diffused from the colony sites. Thus, cormorant colonies have an important impact on benthic communities mainly at a local scale rather than at a larger regional scale. At a larger scale, the net overall impact of cormorants on the nutrient balance of the Baltic Sea may be insignificant. That is, nutrient input from guano may be balanced by the removal of nutrients from the sea through fish predation and the subsequent predation of cormorants by predatory birds (e.g. sea eagles; personal observation), and to a lesser extent by the volatilisation of guano on colony islands.

In addition, owing to fast nutrient intake by periphyton and filamentous algae impacts can vary spatially around a single colony island if guano runoff is not equally distributed e.g. due to topography or variations in wave-exposure [14]. N enrichment was also strongly correlated with cormorant nest density but not with total bird abundance, supporting the idea that it is runoff on a small scale which determines enrichment.

Enrichment factors in marine and aquatic systems are approximately 1–2‰ for herbivores, 2.5‰ for fish, and 3‰ for birds [34], while the actual N signature of cormorants is likely slightly higher than that of the guano excreted. Based upon these enrichment factors, cormorants seem to be feeding at least five levels above producers in this system, likely on piscivorous fish. Recent studies have shown that showing cormorants consume a variety of fish species, including large numbers of benthivores (which consume invertebrates as well as small fish) such as three-spine stickleback (*Gasterosteus aculeatus*), roach (*Rutilus rutilus*), and eelpout (*Zoarces viviparous*) as well as piscivorous predators (mostly perch (*Perca fluviatilis)*), though diet composition varies widely depending on the season and on local fish abundances [38], [39].

The differences encountered in N enrichment between different sections of *F. vesiculosus* thalli within colony sites indicate that enrichment is not only spatially variable, but also temporally variable and relatively short-lived, likely due to fast turnover in the water column. Enrichment was highest in the tips of the thalli (i.e. in the most recently formed parts) and lowest in the base, indicating that more guano-based N had been absorbed in late summer when guano accumulation peaked. Guano is mostly deposited on the colony islands and not directly in the water. Accordingly, rain is necessary for guano runoff. Depending on the periods of intense rainfall, the peak of N enrichment in surrounding waters should vary yearly. Indeed, in 2011, rainfall was highest during the later part of the summer (July-September) than in early summer (May-June) (data from Finnish Meteorological Institute), which can also contribute to the high δ^15^N values found in algal tips. The timing of nutrient enrichment may be very important for its consequences on the biotic community. For example, colonization success of *F. vesiculosus* is highly sensitive to early season enrichment [28], whereas enrichment during the late season (when grazing pressure is highest) may lead to an increased algal palatability [40].

While the stable isotope analysis clearly shows that there is significant N input from cormorant guano, the total N content of samples from colony sites did not differ from that in control sites. This might be due to high N availability and uptake from other sources, therefore anthropogenic eutrophication in the area may mask the impacts of N enrichment from cormorant colonies. Furthermore, total N content increased with δ^15^N signal, particularly in colony sites, but also in control sites, and the source analysis showed that in all colony sites, a large part of algal N uptake originated from cormorant guano. These findings indicate that N from guano is more available for bio-uptake by macroalgae, further supported by the fact that most N in seabird guano is in soluble organic form as uric acid or ammonia [17], [25].

Similar species within the same trophic levels tended to have similar δ^13^C values across both colony and control sites. However, while the periphyton and filamentous algae clustered together, *F. vesiculosus* unexpectedly had an isotopic signature more similar to that of the herbivores tested, and the highest δ^13^C value among all species. Similarly, Kolb et al. [14] found higher δ^13^C values for *F. vesiculosus* than for all other algae and invertebrates along the Swedish Baltic Coast. These differences are likely due to physiological differences in photosynthetic capacity, carbon uptake and metabolism, and structural differences in carbon storage between different functional groups of algae [41], [42], [43], [44]

The variation in stable isotope signature allowed us to use diet mixing analyses. Diet mixing models are a relatively recent development and have been used to determine trophic relationships in several systems, including lakes [45], pelagic communities [46], and tropical rivers [47]. However, several limitations to this approach still exist. Chief among these is the choice of appropriate trophic enrichment factors, which can vary widely between species and even within species in different environments [45], [35], [48], [34]. Furthermore, the true enrichment factors may even vary with the enrichment of the diet [34]. Specific factors for species and their potential food sources can only be specifically determined in controlled feeding experiments and, thus, the diet analyses presented here should be taken as preliminary estimates.

Results from all enrichment factors seem to indicate that *F. vesiculosus* constitutes the primary food source for both *T. fluviatilis and I. balthica*, though the reasons for this likely differ between the two species. *F. vesiculosus* is a preferred microhabitat and food source of *I. balthica* in the Baltic Sea [49], and while *T. fluviatilis* does not feed on adult thalli, it does feed on young germlings [50], [51]. Despite the fact that different fractionation factors yielded slightly different estimates for the amount of consumption of filamentous algae and periphyton, they all showed a decrease in the consumption of filamentous algae along with an increase in periphyton consumption in colony sites. Filamentous algae and periphyton generally benefit from nutrient enrichment, and so the detected decrease in filamentous algal consumption and simultaneous increase in periphyton consumption imply that the grazers prefer periphyton over filamentous algae. Also, the relative availability of periphyton may increase faster than that of filamentous algae with increasing N availability. Both grazers (*T. fluviatilis* and *I. balthica*) are known to feed readily on periphyton growing on *F. vesiculosus* and other substrates [52], [53].

In contrast, Kolb et al. [14] showed that herbivores near colony islands increased their consumption of epiphytic algae, however periphyton was not included in their analysis. Samples for that study were collected in late July, and therefore our differing results may indicate temporal variation in diets as the composition of the algal community shifts over the growing season: there is a bloom of filamentous epiphytic algae in early summer, while germlings of *F. vesiculosus* do not appear until late summer. Also, the *Idotea* samples in that study may have included both *I. chelipes* and juvenile *I. balthica*, which are both smaller than the *I. balthica* sampled for our study in late September, and do not (yet) feed on adult *F. vesiculosus* thalli.

N enrichment in N-limited systems can stimulate algal growth (Worm et al. 2000), but several studies have also shown that N enrichment may also benefit invertebrates. For example, Hemmi and Jormalainen [40] showed that *I. balthica* feeding on N-enriched *F. vesiculosus* had higher growth rates and higher reproductive output (increased size and quantity of eggs) than those fed a control (non-enriched) diet. Similarly, invertebrate abundance and biomass near cormorant colony islands on the east coast of Sweden was higher than near control islands [14]. Accordingly, our study showed significant enrichment in both producers and consumers, therefore it is likely that bottom-up processes are affecting community dynamics and interactions through changes in density and abundance of algae and herbivores.

In evaluating the impacts of cormorant colonies on benthic communities, it is important to consider the potential for interactions with top-down effects. Cormorants are near the top of the benthic pelagic food chain in northern marine communities [54] and the average daily consumption of an adult cormorant is about 0.5 kg of fish [18], [20]. Thus they do not only produce large amounts of guano, but also have the potential to cause trophic cascades through the removal of fish. In general, top-down effects (e.g. trophic cascades) may be of equal or greater importance than bottom-up effects (e.g. nutrient enrichment) on coastal benthic producer communities (reviewed in [23]), demonstrating that trophic networks are complex systems regulated at the same time by both the top-down and bottom-up influences.

Overall, results on N enrichment near cormorant colonies across lower trophic levels shown herein represent an assessment of the bottom-up impacts of cormorant colonies on benthic communities. The top-down effects through trophic cascades may be equally or more important and remain to be studied. Understanding of both bottom-up and top-down processes and their potential interactions in community regulation will be of critical importance in predicting the responses of communities to multiple anthropogenic stressors such as eutrophication and overfishing.
